# Technology for reducing distracted driving in developing countries: the level of usage and intention to use in Indonesia

**DOI:** 10.1016/j.heliyon.2022.e11709

**Published:** 2022-11-23

**Authors:** Kefira Sutanto, Ari Widyanti, Gradiyan Budi Pratama, Herman R. Soetisna

**Affiliations:** Department of Industrial Engineering, Faculty of Industrial Technology, Bandung Institute of Technology, Indonesia

**Keywords:** Distracted-driving, Reduction, Technology, Model, Acceptance

## Abstract

Distracted driving is a major cause of car crashes. Considering the dangers of distracted driving, efforts to develop prevention and/or reduction technology are underway. The purpose of this study is to observe the level of usage of distracted-driving-reduction technology in Indonesia and model the intention to use this technology on the technology acceptance model (TAM).

The participants in this study included 418 Indonesian drivers (217 males, 201 females, mean age = 30.96 years), who volunteered to fill out an online questionnaire that the researcher developed, based on the TAM. The questionnaire comprised constructs including subjective norm, perceived usefulness, perceived ease of use, and intention to use, as well as the additional constructs of trust in technology and personal innovativeness. Participants used a five-point Likert scale to record their responses.

The results showed the level of respondents' use of distracted-driving-reduction technology as 88.52%. The most frequently used technology for this purpose was Bluetooth-enabled audio systems. The factors that significantly influenced the intention to use distracted-driving-reduction technology were the subjective norm, perceived usefulness, perceived ease of use, and personal innovativeness. The paper also discusses the implications of the results.

## 1 Introduction

Distracted driving has recently received increased attention. It is any activity that diverts the driver’s attention from driving, including talking or texting on the phone, eating and drinking, talking to the people in the vehicle, or fiddling with the stereo, entertainment, or navigation system. Essentially, it encapsulates anything that diverts one’s attention from safe driving (National Highway Traffic Safety Administration [NHTSA], 2019).

Driving is a complex activity that requires one’s full attention; therefore, any activities that distract the driver have serious consequences for road safety. NHTSA (2020) reports 3142 fatalities in the United States in 2019, due to distraction-affected crashes of which distracted driving continues to be a major cause. Research by the NHTSA (2019) estimates that driver distraction in its various forms contributes to approximately 25% of police-reported crashes. According to Razi-Ardakani et al. (2019), distraction-related factors are the most important contributor to the severity of car crashes. In addition, Shaaban et al. (2020) state that among young drivers, in-vehicle distractions strongly affect crash likelihood. As more wireless communication, entertainment, and driver-assistance systems proliferate in the vehicle market, the rate of distraction-related crashes will very likely increase (Stutts et al., 2001).

The issue of driver distraction has appeared in the literature for years. Overton et al. (2015) review the level of usage, problems, and prevention of distracted driving. They state that people in the United States seem unaware of or prone to underestimating the impact of cell-phone distraction on their driving performance. Hill (2015) report that the prevalence of distracted driving behavior is very high among college students, who have greater confidence in their driving skills and multitasking ability and are more likely to engage in distracted-driving behavior. Wilson and Stimpson (2010) review the trends in fatalities arising from distracted driving in the United States from 1999 to 2008, concluding that the increase in texting volume since 2005 made a major contribution to the alarming rise in distracted-driving-related fatalities. Those studies show that distracted driving happens very frequently and triggers accidents, justifying the investment of effort in reducing its frequency. The large number of research studies on distracted driving also highlights the issue’s importance.

Various devices and activities appear to have the potential to distract the driver and significantly impair driving performance and safety. Driving distractions can be internal or external. Internal distractions arise from in-vehicle devices or activities—for example, mobile phones (Guo et al., 2021; Widyanti et al., 2020), route-guidance systems and navigation technology (Yang et al., 2021; Oviedo-Trespalacios and Watson, 2021), entertainment systems, and such nontechnological distractions as eating and drinking. External distractions occur outside the vehicle, such as road lighting (Robbins and Fotios, 2021) and digital billboards (Sheykhfard and Haghighi, 2020).

The dangers of distracted driving have motivated efforts to develop prevention and/or reduction technology. This technology is important because it engages the drivers' attention. Certain features of cars effectively prevent or reduce distracted driving, some of which can be found in various cars manufactured within the last decade while others in autonomous vehicles the manufacturer offers exclusively. Such technologies include cell-phone blocking (a feature that enables a phone to automatically enter “do not disturb” mode while in the car), screen mirroring (a feature that facilitates the mirroring of the phone screen on the car screen), and Bluetooth-enabled car audio systems (a feature that enables connecting the phone to a car’s audio system, allowing one to perform activities without one’s taking eyes off the road). The last feature is important because visual load impairs lateral control, leading to accidents (Engström et al., 2005; ; Widyanti et al., 2017a; 2017b). furthermore, visual distraction takes drivers' eyes from the road and changes their visual focus from driving, increasing crash risk (Qi et al., 2020). In contrast, audio load causes less distraction risk than visual load, as a modality different from visual attention. It reduces the distracting effects of secondary driving tasks, not the primary task (Ranney et al., 2005). Oviedo-Trespalacios et al. (2019) even propose voluntary smartphone applications that can prevent distracted driving.

However, the extent to which distracted-driving prevention technologies can increase safety depends on the frequency with which the driver encounters such technologies. Very little is currently known about the relative frequency of use of distracted-driving prevention technologies. More importantly, the factors that influence the intention to use such technologies play an important role as well and can optimize the countermeasures to minimize driver distraction.

Oviedo-Trespalacios and Watson (2021) state that very few interventions have been effective in reducing the level of distracted driving. They have included education campaigns, legislation, and law enforcement. However, in most cases, their effectiveness is limited. For example, to avoid police enforcement, drivers can hide their phones (Oviedo-Trespalacios, 2018). Furthermore, Oviedo-Trespalacios and Watson (2021) express support for using psychological theories to assess the acceptance of prevention and reduction technologies when the use of mobile phones while driving is the distraction.

Regarding the intention to use distracted-driving-prevention technology, some theories have taken psychological approaches into account, one of which is the technology acceptance model (TAM) (Davis, 1989). The TAM examines the probability of individual behavior in the context of voluntary system use. Developed and used in numerous technology-acceptance studies, TAM’s perceived usefulness refers to the degree to which a user believes that using a particular system/product would improve his/her performance. Meanwhile, perceived ease of use describes the extent to which a person finds the use of the system or product easy.

The TAM has been applied in various areas, including mobile services (Zarmpou et al., 2012), e-commerce (Zhang et al., 2012), and fashion products (Yang and Shih, 2020). Most importantly, the TAM has been used in examining the acceptance of car-related technologies, such as autonomous vehicles (Herrenkind et al., 2019; Yuen et al., 2020) and mobile navigation applications (Yang et al., 2021). In addition, Huang (2021) applied the TAM for potential designers of unmanned cars in research on usage intention. While the research does not relate directly related to the study on distracted driving, Huang (2021) shows an example of TAM’s application in examining the acceptance of car-related technologies.

Other factors that may influence the acceptance of distracted-driving prevention technology are trust, personal innovativeness, and subjective norms. Trust is a very relevant factor in the use of new technology. Mayer et al. (1995) define trust in technology as the willingness to put oneself in a vulnerable position with a certain technology, combined with an expectation of a positive result or positive future behavior. The user decides to trust the system, expecting positive experiences with it. Usually, a user associates higher risk or fear with facing a new and unknown system (Bekiaris and Stevens, 2005).

Günthner and Proff (2021) define personal innovativeness as the extent to which a person buys a new product at an earlier stage of the market than other consumers. It can also be an individual’s enthusiasm for engaging with new technology and willingness to accept an innovation earlier than others. High-degree innovation usually involves a more positive attitude toward technology. Such studies find a relationship between personal innovativeness and acceptance of various applications, with regard to perceived usefulness, ease of use, and intention to use. These applications include driver-assistant systems among older adults (Günthner and Proff, 2021), mobile device options (Lee, 2019), and e-commerce (Bigne-Alcaniz et al., 2008).

A subjective norm can be a person’s perception of what people important to them think they should or should not do regarding the behavior in question (Rouibah and Abbas, 2010). In addition, according to Venkatesh and Davis (2000), a subjective norm has a direct compliance effect when an individual perceives a social actor wanting the individual to perform a certain behavior and having the ability to reward the behavior or punish non-performance.

Indonesia is one of the developing countries suffering from a high number of accidents due to distracted driving, with 100,028 accidents and 23,529 fatalities in 2020. Thus, in general, two or three people die every hour, due to traffic accidents in Indonesia. This is quite alarming, especially as 60% of these accidents correlate with driving behavior, including distracted driving. Indonesia has anti-distracted-driving laws, namely, 2009 UU No. 22, Section 283, which states that anyone who engages in reckless or distracted driving on the road will receive a maximum of three months' imprisonment or a maximum fine of Rp 750,000 (USD 53). However, the number of accidents remains high, indicating that this law is not very effective.

The present study intends to observe the level of usage of distracted-driving-reduction technology in Indonesia and model the intention to use this technology on the extended TAM. Researchers expect that perceived usefulness, perceived ease of use, the subjective norm, trust in technology, and personal effectiveness will influence the use of distracted-driving-prevention technology. Governments can use the results of this study to craft rules and regulations for utilizing such technology to ultimately reduce traffic accidents. Moreover, the results can give a general idea of the use of this technology in developing countries. The resulting model can also provide information about significant factors that influence the intention to use distracted-driving-reduction technology, to facilitate interventions that can increase its use to minimize accidents.

## 2 Methods

### 2.1 Respondents

The participants in this study included 418 Indonesian car drivers, who voluntarily filled out an online questionnaire (217 males, 201 females; mean age = 30.96 years; SD = 12.02 years). Most respondents (44.74%) drive about 6–20 km per day. The questionnaire was posted through a messaging application, to several communities (e.g., car communities) to reach respondents. The respondents were chosen using convenience sampling and required to have held a driver’s license for a minimum of one year, to ensure that they were qualified to respond to the questions. The number of respondents this study required was 385, an unknown population with a confidence interval of 95% and an error margin of 5%.

### 2.2 Questionnaire

**Figure 1:**
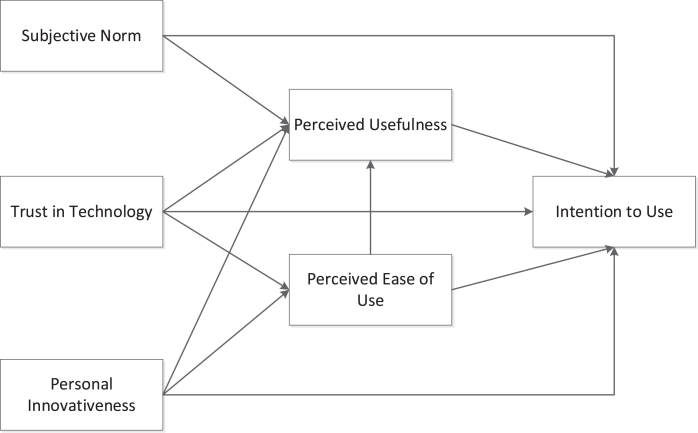
The conceptual model of the intention to use distracted-driving-reduction technology (Günthner and Proff, 2021).

This study implemented a self-developed questionnaire, consisting of section 1 with questions on the respondent’s demographic data and section 2 with the items relevant to the extended theory of acceptance model (TAM) (Günthner and Proff, 2021), the model for which appears in Figure 1. A five-point Likert scale was used for the responses (1 = strongly disagree, 5 = strongly agree). Two statements each represented the constructs of intention to use, perceived usefulness, perceived ease of use, subjective norm, and trust in technology. An example of the statements of intention to use is, “I will use distracted-driving-reducing technology,” The personal innovativeness construct consisted of four statements; for example, “I like to experiment with new information technologies.”

The questionnaire used three examples of distracted-driving-reducing technology: phone mirroring, Bluetooth-enabled audio system, and cell-phone blocking. Phone mirroring is a technology to display the smartphone application on the car dashboard display. The Bluetooth-enabled audio system can receive a phone call through the in-car audio system using a Bluetooth connection. Cell-phone blocking partially inactivates the gadget while the car is moving.

Presented in Bahasa Indonesia, the questionnaire translation process followed a back-translation procedure. First, two Indonesian bilinguals translated the questionnaire into Bahasa Indonesian, and the best Indonesian version was chosen after discussion with a human-factors expert. Then, the Indonesian version was back-translated into English by a third bilingual who had not seen the original English version. The original and back-translated English versions were compared on both the content and context. Completing the questionnaire required on average 10 min. Five lucky respondents received the incentive of 100,000 rupiahs (6.95 $ US).

### 2.3 Data analysis

All respondents' data is included in the analysis. Because all questions required an answer to submit the questionnaire, there was no possibility of missing responses. The presentation of demographic data of the respondents and the intention to use distracted-driving-reduction technology used descriptive statistics. A complex relationship exists among the various determinants of intention to use. To analyze the influence of each construct, structural equation modeling (SEM) was applied. Considering the sample size, the AMOS software was used to implement a covariance-based SEM approach. SEM has the advantage of accounting for measurement error, to represent a reliable construct variance. The exogenous variables of the model consist of “subjective norm,” “trust in technology,” and “personal innovativeness.” The endogenous variables of the model consist of “perceived usefulness,” “perceived ease of use,” and “intention to use.” These variables include 14 observed variables from self-reported statements.

### 2.4 Research context

Indonesia is a developing country with a high number of traffic accidents. There is a huge disparity in income between urban and rural areas. Internet use in Indonesia occurs in 76.8% of the total population.

## 3 Results and analysis

Respondents' level of usage and intention to use distracted-driving-reduction technology appear in Tables 1 and 2.

**Table 1:** Prevalence of distracted-reduction technology used by respondents.

The use of distracted-driving reduction technology	Percentage of respondents (N = 418)
Use the technology	88.52%
Bluetooth enabled audio systems	76.56%
Phone mirroring	21.53%
Cellphone blocking	13.88%
Not use	11.48%

**Table 2:** Level of usage of distracted-driving-reduction technology by demographics.

Demographic data		Percentage of use	Differences of distracted-driving reduction technology’ use based on the referred demographic data
Overall		88.52%	-
Gender	Male Female	90.32% 86.57%	*χ*^*2*^ (1) = 1.448, *ρ* = 0.229
Age	Young (18–44 years old) Old (more than 44 years old)	89.91% 82.72%	*χ*^*2*^ (1) = 3.326, *ρ* = 0.068
Living area	Java (urban area) Outside Java (rural area)	88.53% 88.37%	*χ*^*2*^ (1) = 0.001, *ρ* = 0.975
Occupation	Employee Student Housewives	87% 93% 77%	*χ*^2^ (2) = 7.744, *ρ* = 0.021∗
Proper knowledge of the distracted-driving-reduction technology prior to study?	Yes No	93.09% 81.98%	*χ*^2^ (1) = 12.298, *ρ* = 0.000∗

∗ Significantly different at *α* = 0.05.

**Figure 2:**
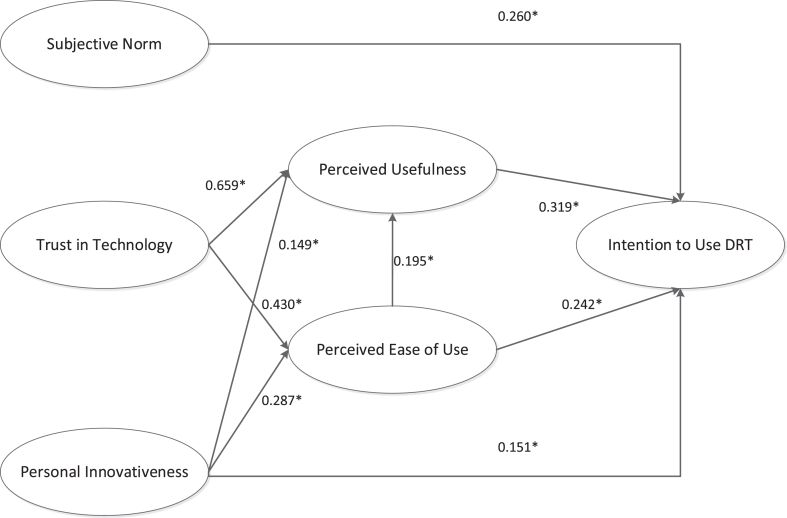
The empirical model of the intention to use distracted-driving-reduction technology.

The model of the intention to use distracted-driving reduction technology appears in Figure 2.

The quality and the goodness-of-fit of the empirical model appear in Tables 3 and 4, respectively.

**Table 3:** The quality of the empirical model.

Construct	Item	Code	Loading factor	Construct reliability	Cronbach’s Alpha	AVE
Intention to use	I will use distracted-driving reducing technology	ITU1	0.867	0.792	0.787	0.657
	I can imagine having the distracted-driving reducing technology in my vehicle	ITU2	0.750			
Perceived usefulness	I think distracted-driving reducing technology is a good idea	PU1	0.875	0.873	0.873	0.775
	I think distracted-driving reducing technology is useful	PU2	0.885			
Perceived ease of use	It would be easy for me to learn how to use distracted-driving reducing technology	PEOU1	0.820	0.746	0.742	0.596
	I do not see any major problems in operating the distracted-driving reducing technology	PEOU2	0.721			
Subjective norm	People who are important to me would appreciate it if I used distracted-driving reducing technology	SN1	0.901	0.900	0.900	0.819
	People who influence my behavior would appreciate it if I used distracted-driving reducing technology	SN2	0.908			
Trust in technology	Distracted-driving reducing technology is technically reliable	TIT1	0.864	0.815	0.813	0.688
	I can trust distracted-driving reducing technology	TIT2	0.794			
Personal innovativeness	I am interested in new products	PI1	0.791	0.830	0.827	0.552
	I like to experiment with new information technologies	PI2	0.833			
	I regularly keep an eye out for new products	PI3	0.709			
	I am usually the one who informs others about new products	PI4	0.621

**Table 4:** Goodness of fit of the model.

Goodness of fit indicator	Value	Fit category	Cut-off Value
Chi-square	*χ*^*2*^ (63) = 209.22, *ρ* = 0.000		
RMSEA	0.075	Acceptable fit	<0.05 or <0.08 (Hair et al., 2010)
CFI	0.954	Acceptable fit	CFI >0.9 (Hair et al., 2010)
SRMR	0.049	Acceptable fit	<0.08 (Hu and Bentler, 1999)

## 4 Discussion and conclusions

This study aimed to observe the level of usage of distracted-driving-reduction technology and to model the intention to use it. The results showed the level of usage of such technology use among the respondents to be 88.52%. The most used distracted-driving-reduction technology was Bluetooth-enabled audio systems. The only demographic data that presented significant differences in the use of distracted-driving-reduction technology were occupation and proper knowledge about the technology. The factors that significantly influenced the intention to use distracted-driving-reduction technology were the subjective norm, perceived usefulness, perceived ease of use, and personal innovativeness.

As Table 1 shows, most participants reported using the distracted-driving-reduction technology, and about two-thirds reported using Bluetooth-enabled audio systems. Only a small proportion of participants used phone-mirroring and cell-phone-blocking technologies. The demographic data of distracted-driving reduction technology usage appear in Table 2. Almost all participants with prior knowledge of the distracted-driving-reduction technology reported having used the technology while driving at some time.

Following the two-step approach to guarantee the fit of the model and test the empirical data (Anderson and Gerbing, 1988), reliability and validity analyses were conducted and appear in Table 3. The construct “reliability” met the cut-off value of 0.6, Cronbach’s Alpha was constantly above 0.7, and the average variance extracted (AVE) performed constantly above 0.5. With the validity and reliability confirmed, fit indices tests could be conducted. Table 4 shows that the model was a good fit to model the intention to use distracted-driving-reduction technology. Running the model brought us to the new model that Figure 2 shows, which implied the hypothesized paths were significant, except for trust in technology as a direct variable associated with intention to use (p = 0.918) and perceived usefulness as an intervening variable between subjective norm and intention to use (p = 0.782).

A significant difference appeared in the use of distracted-driving-reduction technology by occupation; students showed the highest level of usage. This, along with the item “proper knowledge of the technology,” are demographic factors differentiating the use of distracted-driving-reduction technology. The questionnaire presented brief information about distracted driving and descriptions of the distracted-driving-reduction technologies (including function and an example). If respondents understand the concept before reading the information in the questionnaire, the respondent declares having proper knowledge of the technology prior to joining the study. The finding implies that students, who are generally equipped with proper information and well educated in transportation safety, tend to use technologies that support safety.

There were slight differences in the intention to use distracted-driving-reduction technology between genders (males exhibited slightly greater intention to use the technology than females), ages (younger people exhibited slightly higher intention to use the technology than older people), and living areas (people living in urban areas exhibited slightly higher intention to use the technology than people living in rural areas). This result highlights the importance of considering these demographic data in further research on distracted-driving-prevention technology.

The statistical model shows that the model has high validity and reliability. The goodness-of-fit of the model is also apparent. Therefore, the model can explain the intention to use distracted-driving-reduction technology quite well. Furthermore, the model shows that subjective norm, perceived usefulness, perceived ease of use, and personal innovativeness are the factors that influence the intention to use distracted-driving-reduction technology.

Regarding subjective norms, the results of this study are in line with those of other studies on technology acceptance, such as the research on acceptance of driver-assistance systems (Günthner and Proff, 2021) and mobile-phone applications (Oviedo-Trespalacios and Watson, 2021). This may be because peers and family play an important role in influencing the intention to use distracted-driving-reduction technology.

The perceived ease of use and perceived usefulness influencing the intention to use distracted-driving-reduction technology was expected. According to Davis (1989), generating the perception that a product is useful and easy to use triggers willingness to use the product. Moreover, predictably, personal innovativeness was found to significantly influence the intention to use distracted-driving-reduction technology, either directly or with perceived usefulness or perceived ease of use as an intervening mediator variable. This result also aligns with the results of previous studies, including work by Günthner and Proff (2021), who found a significant effect of perceived usefulness and perceived ease of use on the intention to use driver assistance systems. They also argue that those variables were similarly important for both younger and older users.

Trust in technology did not significantly influence the intention to use distracted-driving-reduction technology. However, this influence was significant when factoring in perceived ease of use or perceived usefulness as an intervening variable. This may be because trust alone is not enough to elicit the intention to use the technology. However, trust can trigger perceived ease of use and perceived usefulness, causing one to be eager to use the technology. This result is also consistent with the results of previous studies on the acceptance of vehicle and information technologies. Choi and Ji (2015) report that trust exhibited a significant effect on perceived usefulness of an autonomous vehicle, perceived usefulness and attitude toward e-commerce technology (Wu et al., 2011), perceived usefulness and ease of use of mHealth technology (Schnall et al., 2015), and perceived usefulness of telebanking technology (Alalwan et al., 2016).

This study has several notable limitations. First, using an online questionnaire could have prevented several drivers from filling out the questionnaire, due to limitations regarding Internet access. Expanding the study through paper-based surveys will enhance the analysis. Second, the distracted-driving-reduction technology addressed here is limited to technology commonly used among the respondents. Expanding the variety of technology will further enrich the analysis. Third, the question about the usage level of technology for reducing distracted driving is limited as to whether the respondents ever used the technology. Further analysis of the frequency of use of the technology will enrich the study.

## 5 Conclusion

This study is the first to observe the intention to use distracted-driving-reduction technologies in Indonesia. Such studies are essential for helping the government craft and implement rules and regulations for reducing distraction among drivers, which will ultimately reduce traffic accidents in Indonesia. For example, the Indonesian government can set regulations that obligate the use of distracted-driving reduction and ensure the effectiveness of the regulation, according to the model, by providing proper information on these technologies, particularly emphasizing their usefulness and ease of use.

This study also provides a glimpse into the condition of distracted-driving-reduction technology, particularly in developing countries. Lessons learned from this study for other developing countries include that the use of distracted-driving-reduction technology should be well-observed as an effort to reduce accidents. To increase the use of such technology, proper knowledge about it, especially its usefulness and ease of use, can be enhanced.

## Declarations

### Author contribution statement

Kefira Sutantio: Performed the experiments; Analyzed and interpreted the data; Contributed reagents, materials, analysis tools or data; Wrote the paper.

Ari Widyanti & Gradiyan Budi Pratama: Conceived and designed the experiments; Analyzed and interpreted the data; Contributed reagents, materials, analysis tools or data; Wrote the paper.

Herman R. Soetisna: Conceived and designed the experiments; Analyzed and interpreted the data; Contributed reagents, materials, analysis tools or data.

### Funding statement

The authors were supported by 10.13039/501100015689Institut Teknologi Bandung, Indonesia.

### Data availability statement

Data included in article/supp. material/referenced in article.

### Declaration of interest’s statement

The authors declare no conflict of interest.

### Additional information

No additional information is available for this paper.

## Figures and Tables

**Table:** 

Basic Theory	Construct	Item	Questionnaire Item	References
Technology Acceptance Model (Davis, 1989)	Intention to use	ITU1	I will use distracted-driving reducing technology	Davis (1989)
		ITU2	I can imagine having the distracted-driving reducing technology in my vehicle	
	Perceived usefulness	PU1	I think distracted-driving reducing technology is a good idea	Davis (1989)
		PU2	I think distracted-driving reducing technology is useful	
	Perceived ease of use	PEOU1	It would be easy for me to learn how to use distracted-driving reducing technology	Davis (1989)
		PEOU2	I do not see any major problems in operating the distracted-driving reducing technology	
Acceptance Model from Günthner and Proff (2021)	Subjective norm	SN1	People who are important to me would appreciate it if I used distracted-driving reducing technology	Venkatesh et al. (2003)
		SN2	People who influence my behavior would appreciate it if I used distracted-driving reducing technology	
	Trust in technology	TIT1	distracted-driving reducing technology is technically reliable	McKnight et al. (2011)
		TIT2	I can trust distracted-driving reducing technology	
	Personal innovativeness	PI1	I am interested in new products	Lee (2019)
		PI2	I like to experiment with new information technologies	
		PI3	I regularly keep an eye out for new products	
		PI4	I am usually the one who informs others about new products	

**Table:** 

No.	Construct	Item	Question	Answer
1.	Intention to use	ITU1	*Saya akan menggunakan DRT*	1) *Sangat tidak setuju* 2) *Tidak setuju* 3) *Netral* 4) *Setuju* 5) *Sangat setuju*
		ITU2	*Saya membayangkan memiliki DRT di kendaraan saya*	1) *Sangat tidak setuju* 2) *Tidak setuju* 3) *Netral* 4) *Setuju* 5) *Sangat setuju*
2.	Perceived usefulness	PU1	*Menurut saya DRT adalah ide yang bagus.*	1) *Sangat tidak setuju* 2) *Tidak setuju* 3) *Netral* 4) *Setuju* 5) *Sangat setuju*
		PU2	*Menurut saya DRT bermanfaat.*	1) *Sangat tidak setuju* 2) *Tidak setuju* 3) *Netral* 4) *Setuju* 5) *Sangat setuju*
3.	Perceived ease of use	PEOU1	*Akan mudah bagi saya untuk mempelajari cara menggunakan DRT*	1) *Sangat tidak setuju* 2) *Tidak setuju* 3) *Netral* 4) *Setuju* 5) *Sangat setuju*
		PEOU2	*Saya tidak melihat adanya masalah besar dalam mengoperasikan DRT*	1) *Sangat tidak setuju* 2) *Tidak setuju* 3) *Netral* 4) *Setuju* 5) *Sangat setuju*
4.	Subjective norm	SN1	*Orang-orang yang penting dalam hidup saya akan menghargai bila saya menggunakan DRT*	1) *Sangat tidak setuju* 2) *Tidak setuju* 3) *Netral* 4) *Setuju* 5) *Sangat setuju*
		SN2	*Orang yang memiliki pengaruh terhadap perilaku saya akan menghargai bila saya menggunakan DRT*	1) *Sangat tidak setuju* 2) *Tidak setuju* 3) *Netral* 4) *Setuju* 5) *Sangat setuju*
5.	Trust in technology	TIT1	*Secara teknis, DRT dapat diandalkan*	1) *Sangat tidak setuju* 2) *Tidak setuju* 3) *Netral* 4) *Setuju* 5) *Sangat setuju*
		TIT2	*Saya dapat mempercayai DRT dalam mengurangi gangguan perhatian pada saat mengemudi* (distracted-driving reduction)*.*	1) *Sangat tidak setuju* 2) *Tidak setuju* 3) *Netral* 4) *Setuju* 5) *Sangat setuju*
6.	Personal innovativeness	PI1	*Saya tertarik pada produk baru*	1) *Sangat tidak setuju* 2) *Tidak setuju* 3) *Netral* 4) *Setuju* 5) *Sangat setuju*
		PI2	*Saya senang bereksperimen dengan teknologi baru*	1) *Sangat tidak setuju* 2) *Tidak setuju* 3) *Netral* 4) *Setuju* 5) *Sangat setuju*
		PI3	*Saya memantau perkembangan produk baru secara rutin*	1) *Sangat tidak setuju* 2) *Tidak setuju* 3) *Netral* 4) *Setuju* 5) *Sangat setuju*
		PI4	*Saya adalah orang yang biasanya memberitahukan orang lain tentang produk baru*	1) *Sangat tidak setuju* 2) *Tidak setuju* 3) *Netral* 4) *Setuju* 5) *Sangat setuju*
